# Sleep matters: the U-shaped relationship between sleep duration and overactive bladder in U.S. adults

**DOI:** 10.3389/fmed.2025.1612280

**Published:** 2025-09-18

**Authors:** Ciyi Guan, Lei Wang, Chao Wang, Yinhua Ma, Yinjiao Chen, Junjun Wu, Hui Pan, Chang Zhang, Haiyuan Song

**Affiliations:** ^1^The Eighth Clinical Medical College of Guangzhou University of Traditional Chinese Medicine, Foshan, Guangdong, China; ^2^Foshan Hospital of Traditional Chinese Medicine, Foshan, Guangdong, China; ^3^The Tenth Clinical Medical College of Guangzhou University of Chinese Medicine, Zhongshan, Guangdong, China; ^4^Zhongshan Hospital of Traditional Chinese Medicine, Zhongshan, China; ^5^Southern Medical University Hospital of Integrated Traditional Chinese and Western Medicine, Guangzhou, China; ^6^Xiangnan University, Hunan, China; ^7^School of Traditional Chinese Medicine, Jinan University, Guangzhou, China; ^8^The First Affiliated Hospital of Hunan University of Medicine, Huaihua, China

**Keywords:** overactive bladder, sleep duration, sleep disorders, U-shaped, NHANES

## Abstract

**Objectives:**

To investigate the independent and combined associations of sleep duration and sleep disorders with the risk of overactive bladder (OAB) and identify threshold effects of sleep duration on OAB.

**Methods:**

Data from the NHANES (2005–2018) were analyzed, including 27,302 adults, among whom 5,601 (20.5%) were diagnosed with OAB. Associations between sleep duration (≤ 6 h, > 6 to < 9 h, ≥ 9 h), sleep disorders, and OAB risk were assessed using multivariable logistic regression, restricted cubic splines (RCS), and smooth curve fitting, adjusting for demographic, socioeconomic, lifestyle, and health-related covariates.

**Results:**

A significant non-linear, U-shaped relationship between sleep duration and OAB risk was observed. Compared to individuals with a sleep duration of 6–9 h, those with short sleep duration (≤ 6 h) had a slightly lower risk of OAB (OR = 0.94), while those with long sleep duration (≥ 9 h) had a significantly higher risk (OR = 2.54). Self-reported sleep disorders independently elevated the risk of OAB (OR = 1.46). RCS analysis identified 6 h of sleep as a critical inflection point.

**Conclusion:**

This study reveals a U-shaped relationship between sleep duration and the risk of overactive bladder (OAB), with both short (≤ 6 h) and long (≥ 9 h) sleep durations increasing OAB risk. These findings highlight the importance of sleep management in OAB care. Behavioral interventions, including sleep hygiene education and bladder training, may help mitigate symptoms and improve patient outcomes.

## 1 Introduction

Overactive bladder (OAB) is a chronic lower urinary tract dysfunction syndrome characterized by symptoms such as urinary urgency, urinary frequency and nocturia, which seriously affects patients’ quality of life and psychological health (1). The global prevalence is about 10%–16%, and the prevalence of OAB is on the rise with the aging of the population (2). According to epidemiologic estimates, more than 460 million adults worldwide are afflicted by OAB, and its direct medical costs and indirect economic burden are increasing each year, with associated health expenditures projected to increase by 42% by 2030 (3). The pathogenesis of OAB is complex and involves a multifactorial interaction of bladder sensory nerve abnormalities, detrusor overactivity, and imbalance of central nervous system regulation (4). In addition to non-modifiable risk factors such as age and gender, lifestyle factors, such as fluid intake patterns and sedentary habits, have been shown to be strongly associated with symptom exacerbation in OAB (5, 6), suggesting that intervening with these modifiable behavioral factors may be an important direction in the management of OAB.

Behavioral therapy is the first-line treatment strategy for OAB, and studies have shown that lifestyle modifications such as bladder training and fluid management are effective in relieving symptoms in 30%–50% of patients (7, 8). In recent years, more and more studies have begun to focus on the potential impact of sleep on bladder function, which is considered to be a central aspect of circadian rhythm regulation and may play an important role in bladder function through the neuro-endocrine-immune axis (9). Clinical observations have found that abnormal sleep duration is closely associated with increased frequency of nocturia and decreased bladder capacity (10, 11), while sleep disorders such as insomnia and sleep apnea, which are linked to sympathetic activation and can also exacerbate headaches and orofacial pain, may further destabilize the urethral muscle (12–14). In addition, there may be a bidirectional relationship between sleep quality and OAB symptoms: increased frequency of nocturia may lead to sleep fragmentation, while sleep deprivation may exacerbate sensory hypersensitivity of the bladder through elevated levels of inflammatory factors (e.g., IL-6, TNF-α) (15).

However, existing studies have mainly focused on a single sleep dimension, with fewer systematic explorations of the combined effects of sleep duration and sleep disorders. Despite the growing interest in the potential value of sleep health in the management of OAB, there is still a lack of cross-sectional evidence based on large sample populations to elucidate the independent and synergistic effects of sleep duration and sleep disorders on OAB. Therefore, using data from a nationally representative health survey, this study is the first to comprehensively assess the association patterns between sleep duration and sleep disorders and the risk of OAB, aiming to provide a new scientific basis for multidimensional behavioral interventions for OAB. We hypothesize that both short and long sleep durations are associated with an increased risk of OAB, and that this effect is modified by the presence of sleep disorders.

## 2 Materials and methods

### 2.1 Study cohorts

The National Health and Nutrition Examination Survey (NHANES) is a comprehensive cross-sectional study aimed at assessing the health and nutritional profiles of both adults and children across the United States. Utilizing a complex, multistage probability sampling approach, NHANES ensures the collection of data from a representative cohort of the non-institutionalized civilian population. Data acquisition is achieved through a combination of structured household interviews, detailed physical examinations conducted at mobile examination centers, comprehensive laboratory analyzes, and health-related questionnaires (16, 17). All datasets utilized and examined in this study are publicly available and can be accessed via the official NHANES website.^1^

**FIGURE 1:**
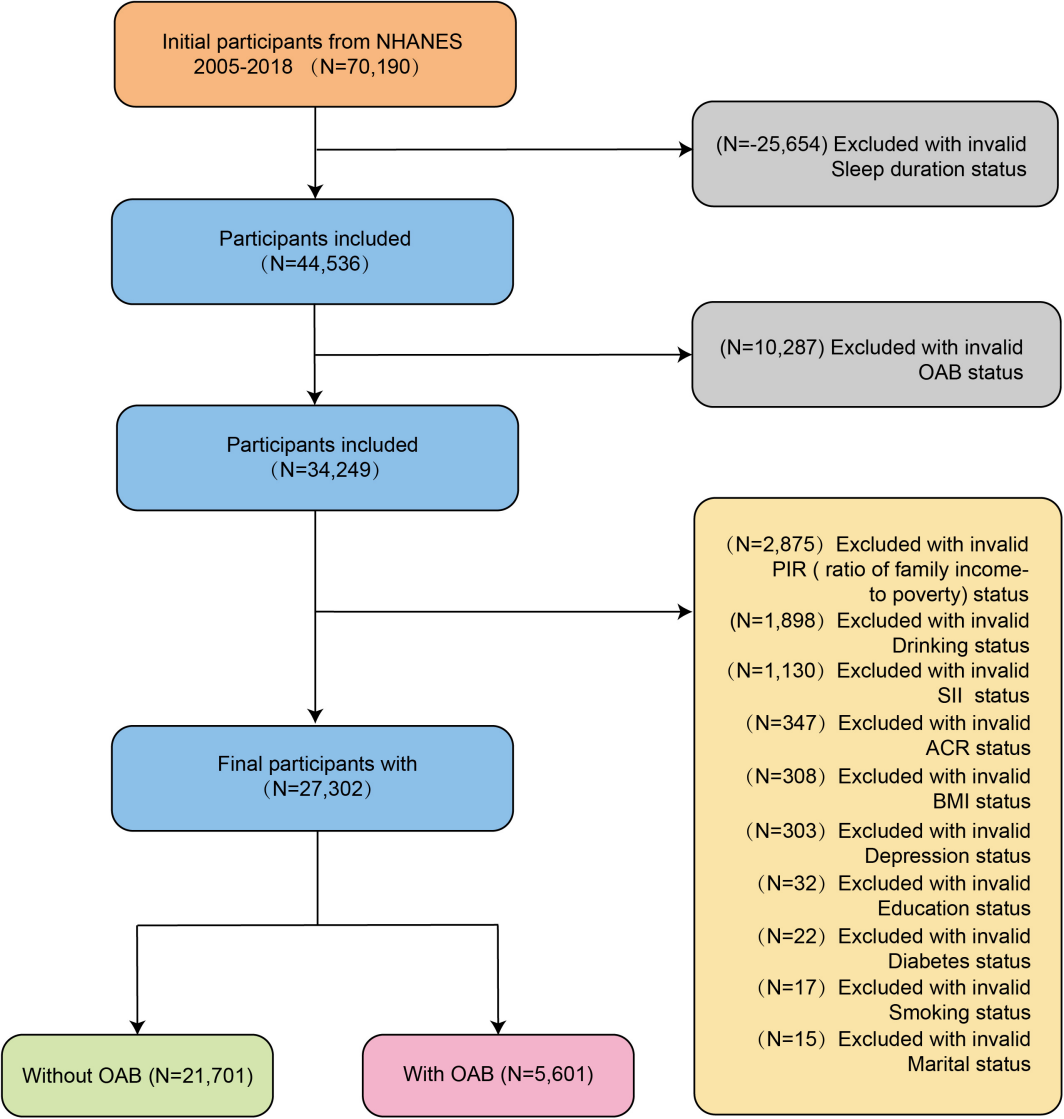
Flowchart of the selection of participants from National Health and Nutrition Examination Survey (NHANES) 2005–2018.

Initially, 70,190 participants (≥ 18 years) were enrolled from seven 2 years cycles of NHANES (2005–2018). After data consolidation, we sequentially excluded 25,654 individuals with invalid sleep duration data, 10,287 with missing OAB status, 2,875 with incomplete poverty-income ratio (PIR), and 1,898 with undocumented drinking status. Further exclusions comprised 1,130 participants lacking systemic inflammation index (SII), 347 with missing albumin-to-creatinine ratio (ACR), 308 with invalid body mass index (BMI), 303 with incomplete depression data, 32 with undocumented education level, 22 with unverified diabetes status, 17 with incomplete smoking records, and 15 with missing marital status. Ultimately, 27,302 eligible adults were included in the final analysis, with 5,601 (20.5%) classified as OAB cases and 21,701 (79.5%) as non-OAB controls (Figure 1).

### 2.2 Ethical approval and participant consent

The data analyzed in this research were sourced from the publicly available NHANES database. The study protocols received formal approval from the Research Ethics Review Board (ERB) of the National Center for Health Statistics (NCHS). Prior to participation, written informed consent was obtained from all individuals involved in the survey. Further details regarding ethical compliance can be accessed at the official NHANES website (see text footnote 1) (17)

### 2.3 Sleep-related variables

Sleep-related variables in this study were derived from the NHANES Sleep Disorders Section. Specifically, sleep duration was assessed using the variable “SLQ012: Sleep hours,” which asked participants, “How much sleep do you usually get at night on weekdays or workdays?” Valid responses ranged from 2 to 14.5 h, with 33 missing entries. Sleep difficulties were assessed using the variable “SLQ050: Ever told doctor had trouble sleeping?” Participants were asked, “Have you ever told a doctor or other health professional that you have trouble sleeping?” Responses included “Yes,” “No,” “Refused,” and “Don’t know.” Entries marked as “Refused” or “Don’t know” were treated as missing data.

To ensure consistency across survey cycles, we used the SLQ012 variable to assess sleep duration in all NHANES cycles from 2005 to 2018. Although this self-reported variable is available in a harmonized format across cycles, the original data collection methods varied. Specifically, during the 2005–2014 cycles, sleep duration was directly reported by participants in whole-hour increments. In contrast, in the 2015–2018 cycles, sleep duration was derived from reported bedtimes and wake-up times, and then rounded to the nearest half hour. While SLQ012 provides a standardized measure across cycles, potential inconsistencies in underlying data collection procedures should be acknowledged as a source of measurement variation.

### 2.4 Assessment of OAB

The diagnosis of OAB hinges on the presence of urge urinary incontinence and nocturia, as defined by clinical criteria. To operationalize this assessment, three core questions from the NHANES Kidney Conditions-Urology questionnaire were utilized:(1) During the past 12 months, have you leaked or lost control of even a small amount of urine with an urge or pressure to urinate and you couldn’t get to the toilet fast enough? (2) How frequently does this occur? (3) During the past 30 days, how many times per night did you most typically get up to urinate, from the time you went to bed at night until the time you got up in the morning? Symptom frequencies were mapped to the Overactive Bladder Symptom Score (OABSS) framework, with nocturia and urgency incontinence scores derived from standardized criteria (Table 1). A composite OABSS score was generated by summing these sub-scores, and participants exceeding a threshold of ≥ 3 were classified as meeting clinical OAB diagnostic criteria (18). According to the scoring criteria of the Overactive Bladder Symptom Score (OABSS), nocturia frequency was mapped to corresponding point values to generate the total OAB score. Specifically, a frequency of 0 episodes per night was scored as 0, 1 episode as 1 point, 2 episodes as 2 points, and 3 or more episodes as 3 points. This conversion method was applied to standardize symptom severity across participants and ensure comparability with established clinical diagnostic thresholds.

**TABLE 1:** Criteria for conversion of symptom frequencies recorded in National Health and Nutrition Examination Survey (NHANES) and Overactive Bladder Symptom (OABSS) scores.

According to NHANES score	According to OABSS score
**Urge urinary incontinence frequency**	**Urge urinary incontinence score**
Never	0
Less than once a month	1
A few times a month	1
A few times a week	2
Every day or night	3
**Nocturia frequency score**	**Nocturia frequency score**
0	0
1	1
2	2
3	3
4	3
5 or score	3

Nocturia frequency scores were assigned as follows based on OABSS criteria: 0 times, 0 points; 1 time, 1 point; 2 times, 2 points; 3 or more times, 3 points.

### 2.5 Independent variables

To control for confounding effects, we included comprehensive covariates in the analysis. Demographic covariates comprised age (years), gender (male/female), and race/ethnicity (Mexican American, Other Hispanic, non-Hispanic Black, non-Hispanic White people, and Other Race). Socioeconomic factors included the PIR, educational attainment (less than high school, high school/GED, above high school), and marital status (married/living with partner, widowed/divorced/separated, or never married). Lifestyle covariates encompassed smoking status (never, former, current smoker) and drinking behavior (never, low, or heavy drinker). Health-related covariates incorporated hypertension (yes/no), diabetes mellitus (yes/no), BMI (kg/m^2^), ACR (mg/g), SII, depression status (yes/no), and sleep trouble (yes/no). These variables were selected based on their established associations with OAB pathophysiology and metabolic health, and were rigorously adjusted for in multivariable models to mitigate confounding bias.

### 2.6 Statistical analysis

Analyzes were conducted using Empower version 4.2 (X&Y Solutions, Inc., Boston, MA, United States) and R version 3.4.3 (R Foundation for Statistical Computing). To account for NHANES’s complex multistage probability sampling design, sampling weights were incorporated in baseline population characterization and multivariable logistic regression analyzes, with variance estimation adjusted for clustering and stratification. Restricted cubic spline (RCS) analysis was used to examine the non-linear relationship between sleep duration and OAB risk. The RCS method allowed for the flexible modeling of the association by allowing for different effects at different levels of sleep duration. A segmented linear regression model was then applied to identify the inflection point for sleep duration, where the relationship between sleep duration and OAB risk changed. This model helped identify the threshold effect of sleep duration on OAB risk. However, subgroup analyzes and smooth curve fitting were performed without weighting to avoid potential overadjustment in stratified samples.

Baseline characteristics were summarized as weighted means ± standard errors for continuous variables and weighted frequencies with percentages for categorical variables. Group differences between OAB and non-OAB participants were evaluated using weighted chi-square tests (categorical variables) and weighted *t*-tests (continuous variables).

Multivariable logistic regression models were developed in Empower to assess associations between sleep duration [categorized as ≤ 6 h, > 6 to < 9 h (reference), and ≥ 9 h] (19), self-reported trouble sleeping (yes/no), and OAB risk. Three sequential models were constructed: Model 1 (unadjusted), Model 2 (adjusted for sex, age, and race/ethnicity), and Model 3 (fully adjusted for sex, age, race/ethnicity, education level, marital status, PIR, BMI, ACR, SII, smoking status, drinking status, hypertension, and depression). Adjusted odds ratios (ORs) with 95% confidence intervals (CIs) and trend *P*-values (calculated via the Cochran-Armitage test) were reported to evaluate dose-response relationships.

## 3 Results

### 3.1 Characteristics of the study population

This cross-sectional study included 27,302 U.S. adults (NHANES 2005–2018), with 5,601 (20.5%) diagnosed with OAB. Baseline characteristics demonstrated significant differences between the OAB (*N* = 5,601) and non-OAB groups (*N* = 21,701). The prevalence of OAB was highest in the group with sleep duration 6–9 h (78.98%), followed by the group with sleep duration ≤ 6 h (10.82%) and the group with sleep duration ≥ 9 h (10.20%). The OAB group had a higher proportion of females (55.45% vs. 49.82%, *P* < 0.001) and non-Hispanic White people (23.76% vs. 20.22%). Marital status analysis revealed lower rates of being married/cohabitating (56.76% vs. 61.04%) but higher rates of widowhood/divorce/separation (27.12% vs. 20.81%) in the OAB group (all *P* < 0.001). Educationally, OAB participants exhibited a higher prevalence of less than high school education (25.62% vs. 22.93%). Chronic disease profiles showed higher diabetes prevalence (21.59% vs. 10.94%) but lower hypertension rates (42.24% vs. 34.20%) in the OAB group. Psychosocial factors indicated elevated rates of sleep trouble (32.48% vs. 24.13%) and depression (15.05% vs. 7.14%) among OAB individuals (all *P* < 0.001). Behaviorally, never drinkers were more common in the OAB group (38.37% vs. 28.42%). The OAB group was younger (48.51 ± 17.64 vs. 52.72 ± 17.87 years), had higher BMI (29.97 ± 7.31 vs. 29.05 ± 6.87 kg/m^2^), and elevated SII (584.72 ± 4 11.45 vs. 534.12 ± 377.03) and ACR (64.02 ± 456.64 vs. 40.64 ± 287.18 mg/g) (all *P* < 0.001). These findings underscore significant associations between OAB and demographic disparities, metabolic profiles, and psychosocial-behavioral factors (Table 2).

**TABLE 2:** Baseline characteristics of the study participants.

Characteristics	OAB (*N* = 165,725,969)		*P*-value
	No	Yes	
	(*N* = 138,112,181)	(*N* = 276,137,88)	
**Gender (%)**			< 0.001
Male	50.18%	44.55%	–
Female	49.82%	55.45%	–
**Race (%)**			< 0.001
Mexican American	15.28%	14.66%	–
Other Hispanic	8.97%	9.53%	–
Non-Hispanic Black	44.72%	42.81%	–
Non-Hispanic White people	20.22%	23.76%	–
Other race	10.81%	9.23%	–
**Marital status (%)**			< 0.001
Married or living with a partner	61.04%	56.76%	–
Widowed/divorced/separated	20.81%	27.12%	–
Never married	18.15%	16.12%	–
**Education (%)**			< 0.001
Less than high school	22.93%	25.62%	–
High school or GED	22.71%	23.57%	–
Above high school	54.36%	50.81%	–
**Diabetes mellitus (%)**			< 0.001
No	89.06%	78.41%	–
Yes	10.94%	21.59%	–
**Smoking status (%)**			< 0.001
Never smoker	54.69%	50.71%	–
Former smoker	24.27%	29.07%	–
Current smoker	21.04%	20.23%	–
**Drinking status (%)**			< 0.001
Never drinker	28.42%	38.37%	–
Low drinker	9.20%	8.46%	–
heavy drinker	62.38%	53.17%	–
**Hypertension (%)**			< 0.001
No	65.80%	57.76%	–
Yes	34.20%	42.24%	–
**Depression (%)**			< 0.001
No	92.86%	4,758 (84.95%)	–
Yes	7.14%	843 (15.05%)	–
**Trouble sleeping (%)**			< 0.001
No	75.87%	67.52%	–
Yes	24.13%	32.48%	–
Age (years), mean ± SD	52.72 ± 17.87	48.51 ± 17.64	< 0.001
PIR, mean ± SD	2.57 ± 1.63	2.42 ± 1.59	< 0.001
BMI (kg/m^2^), mean ± SD	29.05 ± 6.87	29.97 ± 7.31	< 0.001
ACR (mg/g), mean ± SD	40.64 ± 287.18	64.02 ± 456.64	< 0.001
SII, mean ± SD	534.12 ± 377.03	584.72 ± 411.45	< 0.001
**Sleep duration**			< 0.001
> 6, < 9	85.20%	78.98%	–
≤ 6	11.09%	10.82%	–
≥ 9	3.70%	10.20%	–

### 3.2 Logistic regression analyzes

Multivariate logistic regression analyzes showed a significant association between sleep patterns and the risk of OAB. In a fully adjusted model with > 6 to < 9 h as the reference group, short sleep duration (≤ 6 h) was negatively associated with the risk of OAB (Model 3: OR = 0.94, 95% CI: 0.81–0.98, *P* = 0.032), whereas long sleep duration (≥ 9 h) was associated with a significantly higher risk (Model 3: OR = 2.54, 95% CI: 2.09–3.08, *P* < 0.001). Self-reported sleep disturbances independently increased the likelihood of OAB (Model 3: OR = 1.46, 95% CI: 1.31–1.62, *P* < 0.001) (Table 3).

**TABLE 3:** Odds ratios and 95% confidence intervals for overactive bladder (OAB) according to sleep duration and trouble sleeping.

Characteristics	Model 1		Model 2		Model 3	
	**OR (95% CI)**	***P*-value**	**OR (95% CI)**	***P*-value**	**OR (95% CI)**	***P*-value**
Sleep duration	1.38 (1.33, 1.43)	< 0.001	1.33 (1.29, 1.38)	< 0.001	1.34 (1.29, 1.39)	< 0.001
> 6, < 9	Ref	–	Ref	–	Ref	–
≤ 6	1.05 (0.92, 1.20)	0.4483	0.87 (0.76, 1.00)	0.0459	0.94 (0.81, 0.98)	0.032
≥ 9	2.97 (2.59, 3.41)	< 0.001	2.43 (2.01, 2.94)	< 0.001	2.54 (2.09, 3.08)	< 0.001
**Trouble sleeping**						
No	Ref	–	Ref	–	Ref	–
Yes	1.45 (1.32, 1.61)	< 0.001	1.50 (1.35, 1.66)	< 0.001	1.46 (1.31, 1.62)	< 0.001

Model 1: no covariates were adjusted. Model 2: adjusted for gender, race and age. Model 3: adjusted for gender, race, age, education level, marital status, poverty income ratio (PIR), body mass index (BMI), albumin-to-creatinine ratio (ACR), systemic inflammation index (SII), smoking status, drinking status and hypertension. In sensitivity analysis, sleep duration was converted from a continuous variable to a categorical variable (> 6 to < 9, ≤ 6, ≥ 9).

### 3.3 Smooth curve fitting and threshold effect analysis

**FIGURE 2:**
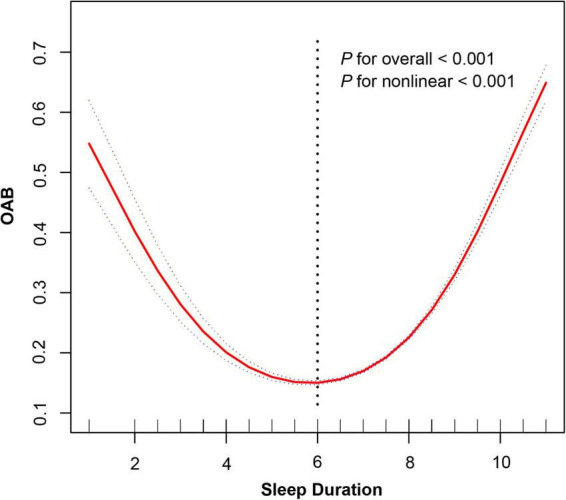
Non-linear relationship between sleep duration and overactive bladder risk.

Restricted cubic spline analysis (Figure 2) revealed a significant non-linear relationship between sleep duration and OAB risk (*P* for non-linearity < 0.001). After adjusting for covariates including gender, race, age, education level, marital status, PIR, BMI, ACR, SII, smoking status, drinking status, and hypertension, a U-shaped association was observed. Specifically, for each additional unit of sleep duration (≤ 6 h), the risk of OAB decreases by 38% (OR = 0.62, 95% CI: 0.59–0.66, *P* < 0.001), whereas prolonged sleep duration (> 6 h) increases the risk by 60% (OR = 1.60, 95% CI: 1.56–1.65, *P* < 0.001). The inflection point was identified at 6 h using a two-segment linear regression model (Log-likelihood ratio test, *P* < 0.001) (Table 4).

**TABLE 4:** Threshold effect analysis of sleep duration on overactive bladder (OAB).

Variable	OR	95% CI	*P-*value
Sleep duration	1.28	(1.25, 1.30)	< 0.001
Inflection point			
< 6	0.62	(0.59, 0.66)	< 0.001
> 6	1.60	(1.56, 1.65)	< 0.001
*P* for log-likelihood ratio test	–	–	< 0.001

Adjusted for gender, race, age, education level, marital status, poverty income ratio (PIR), body mass index (BMI), albumin-to-creatinine ratio (ACR), systemic inflammation index (SII), smoking status, drinking status and hypertension.

### 3.4 Subgroup analysis

**FIGURE 3:**
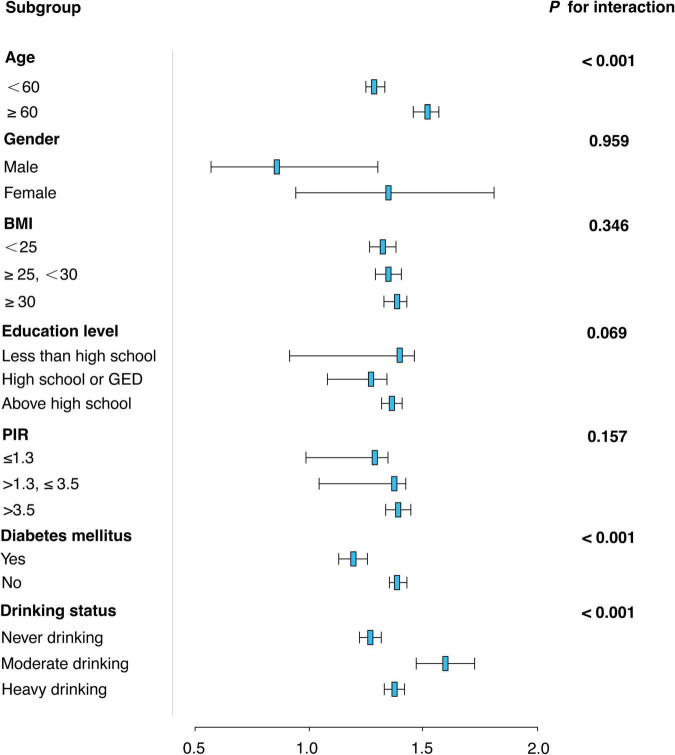
Subgroup analysis of the association between sleep duration and overactive bladder (OAB) risk.

Figure 3 presents a subgroup analysis of the association between sleep duration and OAB risk across various demographic and health-related factors. The analysis shows that the relationship between sleep duration and OAB risk is consistent across most subgroups, including BMI, education level, and PIR (*P* for interaction > 0.05). However, age and diabetes status were significant modifiers of the sleep-OAB association. For age, individuals aged 60 years or older had a stronger association between sleep duration and OAB risk (OR = 1.42, 95% CI: 1.36–1.47, *P* < 0.001) compared to those under 60 (OR = 1.21, 95% CI: 1.18–1.25, *P* < 0.001). For diabetes mellitus, individuals with diabetes showed a weaker association between sleep duration and OAB risk (OR = 1.15, 95% CI: 1.10–1.20) compared to those without diabetes (OR = 1.31, 95% CI: 1.28–1.35). In terms of drinking status, a significant modification of the association was observed. Moderate drinkers had the strongest association between sleep duration and OAB risk (OR = 1.52, 95% CI: 1.39–1.66, *P* < 0.001), while both never drinkers (OR = 1.21, 95% CI: 1.17–1.25, *P* < 0.001) and heavy drinkers (OR = 1.30, 95% CI: 1.26–1.34, *P* < 0.001) also showed significant associations, albeit with different effect sizes.

## 4 Discussion

This study explored the non-linear association between sleep duration and overactive bladder (OAB) risk using NHANES 2005–2018 data. Restricted cubic spline (RCS) analysis revealed a significant non-linear relationship (*P* < 0.001), with an inflection point identified at 6 h. Specifically, among individuals with sleep durations of less than 6 h, the risk of OAB decreased as sleep duration increased (OR = 0.94). In contrast, prolonged sleep duration (> 6 h) was progressively associated with higher OAB risk (OR = 1.60, 95% CI: 1.56–1.65, *P* < 0.001). This U-shaped pattern was further supported by a segmented linear regression model, which confirmed the threshold effect at 6 h.

While Table 3 presents results from categorical logistic regression based on predefined sleep duration groups, the evidence for a non-linear U-shaped association was primarily derived from the RCS and threshold effect models (Figure 2 and Table 4). These methods allowed for a more flexible and detailed characterization of risk across the full spectrum of sleep duration—capturing trends that may not be evident in categorical comparisons alone.

These findings are consistent with previous studies emphasizing the critical role of sleep quality in OAB risk. For example, Lu et al. reported a significantly higher risk of OAB in individuals with poor sleep patterns (OR = 1.38), a finding consistent with our finding of sleep disruption as a risk factor for OAB (20). In addition, Ge et al. (21) associated sleep disruption with more severe OAB symptoms, while Cheng et al. documented a synchronization of increased sleep disorders with increased prevalence of OAB among men in the United States (22, 23). However, unlike these studies, studies focusing on specific populations, such as Guzelsoy et al. and Clerget et al. have focused on hypoxia-driven OAB in patients with obstructive sleep apnea (OSA) (24). In contrast, our population-based study identified sleep disorders as an independent risk factor for OAB, suggesting that there may be broader mechanisms beyond OSA-associated hypoxia, such as circadian rhythm disruption or autonomic dysfunction.

An innovative aspect of this study was the identification of a threshold of 6 hours of sleep duration, a finding that has not been previously reported in epidemiologic studies. Unlike intervention-based studies, such as Mirzayeva et al. who observed linear improvements in the relief of nocturnal urinary frequency with Briverm treatment and Li et al., who included sleep in a composite index of cardiovascular health (LE8), the non-linear association we found emphasizes the dual role of sleep duration in the pathophysiology of OAB that may reflect differences in biological pathways between short and long sleep duration (20, 25). These new insights expand our understanding of sleep-OAB interactions and highlight the need for personalized interventions targeting sleep quantity and quality in reducing the burden of OAB.

The observed non-linear association between sleep duration and OAB risk may be attributed to different physiologic mechanisms operating at different sleep thresholds. Short sleep duration (< 6 h) may cause sympathetic nervous system (SNS) overactivity, which may paradoxically inhibit urethral muscle overactivity in the acute phase through β3-adrenergic receptor-mediated bladder relaxation, as shown by sleep deprivation in animal models (26). However, chronic sleep restriction may disrupt neurotransmitter homeostasis, particularly serotonin and dopamine, which are critical for regulating bladder afferent signaling and central inhibitory pathways (27, 28). Conversely, prolonged sleep may reflect underlying systemic inflammation or circadian rhythm misalignment. Elevated levels of pro-inflammatory cytokines (e.g., IL-6, TNF-α) associated with excessive sleep have been shown to increase bladder permeability and sensitize afferent nerves, which may exacerbate OAB symptoms (29, 30). Further, circadian rhythm misalignment due to irregular sleep-wake patterns may impair hypothalamic regulation of bladder function, including control of urinary aquaporin-2 channels and nocturnal urine production in the supraoptic nucleus (31–33). These bidirectional mechanisms highlight the complexity of the role of sleep duration in the pathophysiology of OAB.

In addition to the findings on the U-shaped relationship between sleep duration and overactive bladder (OAB), it is important to note that sleep hygiene guidelines generally recommend a minimum of 7 h of sleep per night for optimal health. Consistent with current sleep recommendations, this amount of sleep is essential for restorative functions, including physical recovery and cognitive performance, and may play a role in managing OAB symptoms. Sleep durations shorter or longer than this threshold can potentially disrupt physiological processes, contributing to various health issues, including bladder dysfunction.

Furthermore, the subgroup analyzes shown in Figure 3 revealed a complex relationship between sleep duration and OAB risk, which was significantly moderated by age, diabetes status, and drinking habits. Firstly, the moderating effect of age may be closely related to physiological changes during aging. With aging, the body’s metabolic and neuroregulatory capacity gradually declines, especially in the control of urinary tract and the regulation of nocturnal awakenings. The higher risk of OAB in people over 60 years of age may be due to the circadian clock misregulation and nervous system dysfunction caused by aging (34), which makes the effect of sleep on bladder function more significant. The weakening effect of diabetes on the relationship between sleep and OAB may be related to the dysfunction of autonomic nervous system caused by diabetes. Diabetic patients are often accompanied by neuropathy, especially in the sensory and motor nervous systems of the bladder, resulting in reduced sensitivity and functional regulation of the bladder (35), thereby weakening the effect of sleep on the risk of OAB. In addition, metabolic dysregulation in diabetic patients may further affect the physiological mechanisms of nocturnal urine production and excretion (36). In terms of drinking status, the significant association among moderate drinkers may be related to the effect of alcohol on sleep quality. Alcohol can aggravate OAB symptoms by disrupting the structure of sleep, especially during deep sleep and rapid eye movement (REM) sleep stages (37). The diuretic effect of alcohol may also exacerbate frequent urination at night and increase the risk of OAB (38). However, although the association with OAB was stronger between heavy drinkers and never drinkers, the difference in effect size suggests that drinking frequency and the effects of alcohol on the neuroendocrine system have an important modulatory role (39).

The positive association between sleep disorders and OAB risk may involve interrelated pathways in the neuroendocrine and autonomic nervous systems. Sleep disorders such as insomnia or OSA persistently activate the hypothalamic-pituitary-adrenal (HPA) axis, leading to elevated levels of cortisol and catecholamines, which may directly potentiate the excitability of bladder afferent nerves and the contractility of the forced urethra (40). In OSA, intermittent hypoxia-reoxygenation cycles induce oxidative stress and endothelial dysfunction, which may damage bladder neuromuscular junctions and promote overactivity (41). In addition, sleep fragmentation disrupts thalamo-cortical pathways responsible for regulating bladder sensation during non-rapid eye movement sleep, thereby lowering the threshold of awareness of urinary urgency (42). Neuroimaging studies further suggest that chronic sleep disorders alter functional connectivity between the insular cortex, which is associated with visceral perception, and the prefrontal cortex, which is responsible for inhibitory control, potentially amplifying the importance of bladder signaling (43–45).

This study explored the relationship between sleep duration and OAB while controlling for key confounders. However, several limitations must be considered. First, OAB diagnosis was based on symptomatic criteria, not urodynamic validation, which may lead to misclassification of bladder disorders. Second, self-reported sleep duration is prone to measurement errors, with participants often overestimating or underestimating their sleep by 30–60 min. This could lead to non-differential misclassification, especially in individuals with sleep disorders or chronic conditions. If the error is systematic in the OAB group (e.g., individuals with nocturnal urgency reporting shorter sleep), it may introduce bias. Moreover, although we utilized the harmonized SLQ012 variable to maintain consistency in sleep duration measurement across all NHANES cycles, it is important to acknowledge the underlying heterogeneity in the original data collection methods. Earlier cycles (2005–2014) directly asked participants to report their usual sleep duration in whole hours, whereas later cycles (2015–2018) estimated sleep duration based on reported bedtimes and wake-up times. Although SLQ012 standardizes these inputs, variations in data precision and recall bias may introduce subtle misclassification or measurement error. Future studies using objectively measured sleep duration (e.g., actigraphy) are needed to validate these findings. Additionally, due to the cross-sectional design, causality cannot be determined. OAB may cause nocturnal awakenings and shorter sleep, or longer sleep may result from health deterioration associated with OAB. This reverse causality could explain the “short sleep ? lower OAB risk” relationship observed in our study. Future prospective cohort studies with objective sleep measurements (e.g., actigraphy or polysomnography) and standardized urodynamic assessments are needed to clarify the causal relationships between sleep and OAB.

## 5 Conclusion

This study reveals a significant U-shaped non-linear relationship between sleep duration and the risk of overactive bladder (OAB). Specifically, individuals with sleep durations of less than 6 h exhibited a reduced risk of OAB, while those with prolonged sleep durations (≥ 9 h) had a significantly higher risk. These findings underscore the importance of managing sleep patterns as part of the clinical approach to OAB.

Given the clinical implications, it is crucial to consider both sleep quality and duration when evaluating and managing OAB in patients. Behavioral interventions, such as sleep hygiene education, could be beneficial. Recommendations may include promoting a consistent sleep schedule, reducing caffeine and alcohol intake in the evening, and creating a comfortable sleep environment. Additionally, interventions like bladder training and fluid management can complement sleep-based treatments, potentially reducing the frequency and severity of OAB symptoms. Implementing these personalized sleep and behavioral interventions could significantly improve patient outcomes and enhance the overall management of OAB.

## Data Availability

The raw data supporting the conclusions of this article will be made available by the authors, without undue reservation.

## Footnotes

1
https://www.cdc.gov/nchs/nhanes/index.htm

## References

[1] Abrams P Cardozo L Fall M Griffiths D Rosier P Ulmsten U The standardisation of terminology in lower urinary tract function: report from the standardisation sub-committee of the International continence society. *Urology.* (2003) 61:37–49. 10.1016/S0090-4295(02)02243-4 12559262

[2] Irwin D Milsom I Hunskaar S Reilly K Kopp Z Herschorn S Population-based survey of urinary incontinence, overactive bladder, and other lower urinary tract symptoms in five countries: results of the EPIC study. *Eur Urol.* (2006) 50:1306–14. 10.1016/j.eururo.2006.09.019 17049716

[3] Nambiar A Arlandis S Bø K Cobussen-Boekhorst H Costantini E de Heide M European association of urology guidelines on the diagnosis and management of female non-neurogenic lower urinary tract symptoms. Part 1: diagnostics, overactive bladder, stress urinary incontinence, and mixed urinary incontinence. *Eur Urol.* (2022) 82:49–59. 10.1016/j.eururo.2022.01.045 35216856

[4] Andersson K. Mechanisms of disease: central nervous system involvement in overactive bladder syndrome. *Nat Clin Pract Urol.* (2004) 1:103–8. 10.1038/ncpuro0021 16474523

[5] Sakai M Akai T Takata Y Shimizu S Koyama S Iwamoto T. [Screening test for overactive bladder in a newly developed comprehensive geriatric assessment initiative]. *Nihon Ronen Igakkai Zasshi.* (2013) 50:249–57. 10.3143/geriatrics.50.249 23979249

[6] Park J Lee H Kim Y Norton C Woodward S Lee S. Effectiveness of fluid and caffeine modifications on symptoms in adults with overactive bladder: a systematic review. *Int Neurourol J.* (2023) 27:23–35. 10.5213/inj.2346014.007 37015722 PMC10073005

[7] Babin C Catalano N Yancey D Pearl N Koonce E Ahmadzadeh S Update on overactive bladder therapeutic options. *Am J Ther.* (2024) 31:e410–9. 10.1097/MJT.0000000000001637 37171410

[8] Funada S Yoshioka T Luo Y Sato A Akamatsu S Watanabe N. Bladder training for treating overactive bladder in adults. *Cochrane Database Syst Rev.* (2023) 10:CD013571. 10.1002/14651858.CD013571.pub2 37811598 PMC10561149

[9] Ihara T Mitsui T Nakamura Y Kanda M Tsuchiya S Kira S The time-dependent variation of ATP release in mouse primary-cultured urothelial cells is regulated by the clock gene. *Neurourol Urodyn.* (2018) 37:2535–43. 10.1002/nau.23793 30106187

[10] Choi E Wan E Kwok J Chin W Lam C. The mediating role of sleep quality in the association between nocturia and health-related quality of life. *Health Quality Life Outcomes.* (2019) 17:181. 10.1186/s12955-019-1251-5 31829192 PMC6907224

[11] Matsushima E Otsuka Y Itani O Matsumoto Y Kaneita Y. Association between nighttime urinary frequency and sleep problems among Japanese adolescents. *Int J Urol.* (2022) 29:152–7. 10.1111/iju.14744 34786770

[12] Winkelman W Warsi A Huang A Schembri M Rogers R Richter H Sleep quality and daytime sleepiness among women with urgency predominant urinary incontinence. *Urogynecology.* (2018) 24:76. 10.1097/SPV.0000000000000547 29300259 PMC5909832

[13] Orzeszek S Martynowicz H Smardz J Wojakowska A Bombała W Mazur G Assessment of sleep quality in patients with orofacial pain and headache complaints: a polysomnographic study. *Dent Med Probl.* (2024) 61:549–62. 10.17219/dmp/177008 38832763

[14] Martynowicz H Wichniak A Wiêckiewicz M. Sleep disorders and cardiovascular risk: focusing on sleep fragmentation. *Dent Med Probl.* (2024) 61:475–7. 10.17219/dmp/185395 38517218

[15] Brüning F Noya S Bange T Koutsouli S Rudolph J Tyagarajan S Sleep-wake cycles drive daily dynamics of synaptic phosphorylation. *Sci.* (2019) 366:eaav3617. 10.1126/science.aav3617 31601740

[16] Curtin L Mohadjer L Dohrmann S Kruszon-Moran D Mirel L Carroll M National health and nutrition examination survey: sample design, 2007-2010. *Vital Health Stat.* (2013) 2:1–23. 25090039

[17] Zipf G Chiappa M Porter K Ostchega Y Lewis B Dostal J. National health and nutrition examination survey: plan and operations, 1999-2010. *Vital Health Stat, 1 Programs Collect Proced.* (2013) 56:1–37. 25078429

[18] Zhu S Wang Z Tao Z Wang S Wang Z. Relationship between marijuana use and overactive bladder (OAB): a cross-sectional research of NHANES 2005 to 2018. *Am J Med.* (2023) 136:72–8. 10.1016/j.amjmed.2022.08.031 36150516

[19] Zhu Z Wu S Lin X Wang C Zhou X. Association of sleep duration with serum estradiol concentrations among American men and women: evidence from NHANES 2013-2016. *Int J Endocrinol.* (2025) 2025:7863420. 10.1155/ije/7863420 39957841 PMC11828656

[20] Li Z Liu X Li Y Chen X Liu Z Gao X Association between cardiovascular health and overactive bladder. *Sci Rep.* (2025) 15:5760. 10.1038/s41598-025-90438-w 39962171 PMC11832880

[21] Ge T Vetter J Lai H. Sleep disturbance and fatigue are associated with more severe urinary incontinence and overactive bladder symptoms. *Urology.* (2017) 109:67–73. 10.1016/j.urology.2017.07.039 28826875 PMC5669629

[22] Cheng Y Chen T Zheng G Song Z Zhang G Rao X Prevalence and trends in overactive bladder among men in the United States, 2005-2020. *Sci Rep.* (2024) 14:16284. 10.1038/s41598-024-66758-8 39009696 PMC11251073

[23] Clerget A Kanbar A Abdessater M. [urinary tract symptoms and erectile dysfunction in obstructive sleep apnea: systematic review]. *Prog Urol J Assoc Frurol Soc Frurol.* (2020) 30:1069–77. 10.1016/j.purol.2020.07.244 32830023

[24] Guzelsoy M Gunes A Coban S Turkoglu A Onen E Ocakoglu G Frequency of overactive bladder (OAB) and the factors affecting it in patients with obstructive sleep apnea syndrome (OSAS). *Urologia.* (2023) 90:58–67. 10.1177/03915603221078263 35188003

[25] Mirzayeva N Forst S Passweg D Geissbühler V Simões-Wüst A Betschart C. Bryophyllum pinnatum and improvement of nocturia and sleep quality in women: a multicentre, nonrandomised prospective trial. *Evid Based Complement Altern Med Ecam.* (2023) 2023:2115335. 10.1155/2023/2115335 36798727 PMC9928503

[26] Irwin M Olmstead R Carroll J. Sleep disturbance, sleep duration, and inflammation: a systematic review and meta-analysis of cohort studies and experimental sleep deprivation. *Biol Psychiatry.* (2016) 80:40–52. 10.1016/j.biopsych.2015.05.014 26140821 PMC4666828

[27] Kim Y Chen L McCarley R Strecker R. Sleep allostasis in chronic sleep restriction: the role of the norepinephrine system. *Brain Res.* (2013) 1531:9–16. 10.1016/j.brainres.2013.07.048 23916734 PMC3807856

[28] Opp M Krueger J. Sleep and immunity: a growing field with clinical impact. *Brain Behav Immun.* (2015) 47:1–3. 10.1016/j.bbi.2015.03.011 25849976 PMC4685944

[29] Bakour C Schwartz S O’Rourke K Wang W Sappenfield W Couluris M Sleep duration trajectories and systemic inflammation in young adults: results from the national longitudinal study of adolescent to adult health (add health). *Sleep.* (2017) 40:zsx156. 10.1093/sleep/zsx156 29155987 PMC5806583

[30] Negoro H Kanematsu A Doi M Suadicani S Matsuo M Imamura M Involvement of urinary bladder Connexin43 and the circadian clock in coordination of diurnal micturition rhythm. *Nat Commun.* (2012) 3:809. 10.1038/ncomms1812 22549838 PMC3541943

[31] Möller-Levet C Archer S Bucca G Laing E Slak A Kabiljo R Effects of insufficient sleep on circadian rhythmicity and expression amplitude of the human blood transcriptome. *Proc Natl Acad Sci.* (2013) 110:E1132–41. 10.1073/pnas.1217154110 23440187 PMC3607048

[32] Siegel J. The neurotransmitters of sleep. *J Clin Psychiatry.* (2004) 65:4–7. PMC8761080 15575797

[33] Apostolidis A Wagg A Rahnam A’i M Panicker J Vrijens D von Gontard A. Is there “brain OAB” and how can we recognize it? International consultation on incontinence-research society (ICI-RS) 2017. *Neurourol Urodyn.* (2018) 37:S38–45. 10.1002/nau.23506 29388707

[34] Leng Y Musiek E Hu K Cappuccio F Yaffe K. Association between circadian rhythms and neurodegenerative diseases. *Lancet Neurol.* (2019) 18:307–18. 10.1016/S1474-4422(18)30461-7 30784558 PMC6426656

[35] Stowe T McClung C. How does chronobiology contribute to the development of diseases in later life. *Clin Interv Aging.* (2023) 18:655–66. 10.2147/CIA.S380436 37101656 PMC10124625

[36] Ueda T Yoshimura N Yoshida O. Diabetic cystopathy: relationship to autonomic neuropathy detected by sympathetic skin response. *J Urol.* (1997) 157:580–4. 10.1016/s0022-5347(01)65209-1 8996363

[37] Reeves-Darby J Berro L Hembree H Shaffery J Rowlett J Sharmin D Ethanol-induced rapid eye movement sleep suppression in rats: comparison with subtype-selective GABAA receptor compounds. *J Psychopharmacol.* (2025) 39:744–55. 10.1177/02698811251344682 40539934 PMC12935073

[38] Kallas H Chintanadilok J Maruenda J Donahue J Lowenthal D. Treatment of nocturia in the elderly. *Drugs Aging.* (1999) 15:429–37. 10.2165/00002512-199915060-00003 10641954

[39] Ray L Mackillop J Leggio L Morgan M Hutchison K. Effects of naltrexone on cortisol levels in heavy drinkers. *Pharmacol, Biochem Behav.* (2009) 91:489–94. 10.1016/j.pbb.2008.09.004 18824022 PMC3660224

[40] Buckley T Schatzberg A. On the interactions of the hypothalamic-pituitary-adrenal (HPA) axis and sleep: normal HPA axis activity and circadian rhythm, exemplary sleep disorders. *J Clin Endocrinol Metab.* (2005) 90:3106–14. 10.1210/jc.2004-1056 15728214

[41] Lavie L. Oxidative stress in obstructive sleep apnea and intermittent hypoxia–revisited–the bad ugly and good: implications to the heart and brain. *Sleep Med Rev.* (2015) 20:27–45. 10.1016/j.smrv.2014.07.003 25155182

[42] Griffiths D Fowler C. The micturition switch and its forebrain influences. *Acta Physiol.* (2013) 207:93–109. 10.1111/apha.12019 23164237

[43] Weissbart S Bhavsar R Rao H Wein A Detre J Arya L Specific changes in brain activity during urgency in women with overactive bladder after successful sacral neuromodulation: a functional magnetic resonance imaging study. *J Urol.* (2018) 200:382–8. 10.1016/j.juro.2018.03.129 29630979

[44] Qi J Li B Zhang Y Pan B Gao Y Zhan H Altered insula-prefrontal functional connectivity correlates to decreased vigilant attention after total sleep deprivation. *Sleep Med.* (2021) 84:187–94. 10.1016/j.sleep.2021.05.037 34166985

[45] Griffiths D Tadic S. Bladder control, urgency, and urge incontinence: evidence from functional brain imaging. *Neurourol Urodyn.* (2008) 27:466–74. 18092336 10.1002/nau.20549

